# Biomechanical Analysis of Titanium Dental Implants in the All-on-4 Treatment with Different Implant–Abutment Connections: A Three-Dimensional Finite Element Study

**DOI:** 10.3390/jfb14100515

**Published:** 2023-10-12

**Authors:** Pei-Shuang Wang, Ming-Hsu Tsai, Yu-Ling Wu, Hung-Shyong Chen, Yao-Ning Lei, Aaron Yu-Jen Wu

**Affiliations:** 1 Department of Dentistry, Kaohsiung Chang Gung Memorial Hospital and Chang Gung University College of Medicine, Kaohsiung 833, Taiwan; 2 Department of Mechanical Engineering, Cheng Shiu University, Kaohsiung 833, Taiwan; 3 Center for Environmental Toxin and Emerging-Contaminant Research, Cheng Shiu University, Kaohsiung 833, Taiwan

**Keywords:** implant–abutment connections, all-on-4 treatment, finite element analysis

## Abstract

The type of implant-abutment connection is one of the factors influencing the distribution of occlusal forces. This study aims to investigate the biomechanical performance of the mandibular all-on-4 treatment with different implant–abutment connections. Two connection types with 30° abutments and 18-mm implant fixtures were chosen for the posterior implants of the all-on-4 assembly. For the external hexagon connection (EHC) group, the implants with 4 mm in diameter were used. For the internal hexagon connection (IHC) group, we selected implants with 4.3 mm in diameter. A vertical force of 190 N was applied to the cantilever region. The FEA results indicated that the most stressed region in the two groups was prosthetic screws, followed by multi-unit abutments (MUAs). The lowest values of von Mises stress were both observed on the bone. The peak stress value of the implant screw and implant fixture in the EHC group were 37.75% and 33.03% lower than the IHC group, respectively. For stress distribution patterns, the load force tended to be concentrated at locations where components were interconnected. The EHC and IHC are clinically durable under the tested loading conditions, but the prosthetic screws and MUAs can be the weak point on the posterior implant within the mandibular all-on-four assembly. The peak stress values of implant screw and implant fixture in the EHC groups were lower than the IHC group.

## 1. Introduction

According to an investigation by the Ministry of Health and Welfare of Taiwan that was conducted between 2015 and 2016 [1], the mean number of permanent teeth in Taiwanese individuals aged ≥75 years is 16.72, and the mean percentage of individuals with no remaining teeth is 17.4%. These figures are notably lower than those recorded in a similar survey, the National Health and Nutrition Examination Survey, conducted from 2011 to 2016 in the United States, which focused on individuals aged ≥75 years [2]. In the United States, the average number of permanent teeth and the percentage of individuals with no remaining teeth are 19.5 and 22.5%, respectively. Although oral hygiene has considerably improved in Taiwan over the years, many older individuals still experience edentulism, with nearly one in five older adults being completely edentulous. Complete edentulism can present challenges, and patient satisfaction with conventional complete dentures may be influenced by factors such as denture stability and aesthetics [3]. Additionally, in cases where reconstruction involves implant-retained overdenture or fixed prostheses supported by multiple implants, jawbone atrophy can pose difficulties, particularly in the posterior mandible, where poor bone quality can influence the ideal diameter and length for implant placement [4].

To address these challenges, Maló et al. [5] introduced a treatment approach known as the “all-on-4”. This approach involves the placement of four implants, with two positioned vertically in the anterior region and two angled in the posterior region, which provides support for a full-arched fixed denture. The all-on-4 treatment concept offers several advantages, including avoidance of additional bone grafting surgery, prevention of damage to the inferior alveolar nerve, and a reduced cantilever length. Research has demonstrated the effectiveness and long-term viability of all-on-4 treatment for rehabilitating fully edentulous mandibles. In a longitudinal study [6], the cumulative success rate of implants placed using the all-on-4 treatment was reported to be 91.7% up to an 18-year follow-up period. Additionally, a literature review [7] demonstrated that all-on-4 treatment yields similarly high success rates to those of traditional vertical implants, primarily because of favorable biomechanical factors.

Despite its high success rate, the all-on-4 treatment is associated with biological and mechanical complications, such as peri-implant disease, prosthesis fractures, and loosening of abutment or prosthesis screws, which can negatively affect the overall treatment outcome [6,8]. These complications are primarily associated with occlusal overload [9,10], and excessive occlusal force can lead to marginal bone loss. A comprehensive review of the literature revealed that numerous factors influence the transfer of load at the interface between bone and implant, such as characteristics of the bone–implant interface, the quality and quantity of the surrounding bone, the implant length and diameter, the implant shape, the surface structure of the implant, the loading type, and the material properties of both the implant and the prosthesis [11]. Variations in implant designs, including with respect to geometry and the type of implant–abutment connection, also play a critical role in the distribution of occlusal forces [12]. These design differences can affect the performance and maintenance of implant osseointegration [13] and ultimately contribute to mechanical and biological complications that may compromise treatment outcomes and lead to serious complications, including implant breaks [14] and the need for additional surgery, time, and costs.

Based on the types of implant–abutment connections, implant designs can be categorized into external and internal connections. External connections offer several advantages, including a superior passive fit and greater flexibility in cases involving multiple implants, which simplifies the prosthetic phase [15]. The external hexagon connection is the most widely applied in surgical protocols for the all-on-4 treatment developed by Maló et al. [5]. By contrast, internal connections tend to result in less marginal bone loss and promote more homogeneous stress distribution [16]. However, both types of implant–abutment connections are associated with some drawbacks. External connections are prone to mechanical and biological complications caused by abutment micromovement [17,18], whereas internal connections can present challenges with respect to achieving a precise passive fit with multiple implants, which can lead to problems during the prosthetic phase [19,20].

Maló et al. [6] demonstrated the viability of the all-on-4 treatment, but all treatments used the same type of implant–abutment connection. Another randomized, split-mouth controlled trial [21] explored the use of two types of implant–abutment connections, external and internal hexagon connections, in full-arch rehabilitations. Their findings indicated that both types were associated with high success rates within a 3-year follow-up period; however, possibly because of the small sample size, no significant differences were found with mechanical complications. Research on all-on-4 treatment has begun to focus on the biomechanical performance of the treatment. From a bioengineering perspective, a critical concern with this treatment is minimizing peak bone stress resulting from occlusal loading [22]. Many studies have focused on the effects of the cantilever length [23,24], position, and angulation of posterior implants [25,26]. However, the biomechanical performance of different implant–abutment connections in the mandibular all-on-4 treatment remains unclear.

Therefore, the present study investigated the influences of different types of implant–abutment connections in all-on-four treatment on the biomechanical performance of the treatment. The investigation specifically addressed factors such as peak stress and strain values and their distribution patterns across each component of the all-on-4 assembly and the surrounding bone when subjected to an occlusal load applied to the distal cantilever region. To achieve this, we employed three-dimensional (3D) finite element analysis.

## 2. Materials and Methods

### 2.1. 3D Finite Element Modeling

In this study, we analyzed a representative model of mandibular all-on-4 treatment. This model comprised two anterior implants strategically placed in the anterior region and two posterior implants placed in the premolar region. Additionally, our analysis incorporated all essential components of the all-on-4 assembly, including a custom-made titanium framework (implant bar), multi-unit abutments (MUAs), prosthetic screws, and implant screws.

**Figure 1:**
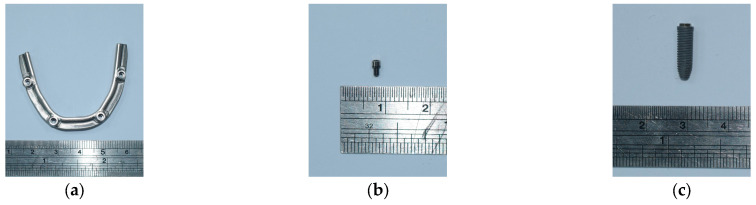

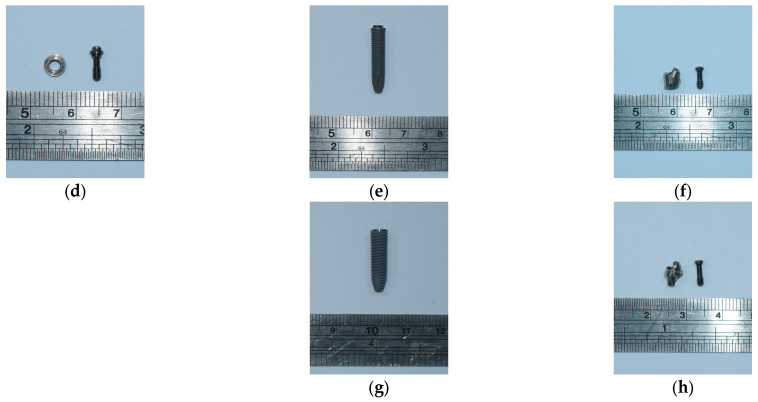
Components of the all-on-4 assembly: (**a**) implant bar; (**b**) prosthetic screw; anterior implant assembly: (**c**) implant fixture, (**d**) abutment, and implant screw; posterior implant assembly in the EHC group: (**e**) implant fixture, (**f**) abutment, and implant screw; posterior implant assembly in the IHC group: (**g**) implant fixture, (**h**) abutment, and implant screw.

The anterior implants in the analyzed model included implants with a diameter of 4 mm and a length of 13 mm (NobelSpeed^TM^ Groovy, Nobel Biocare, Goteborg, Sweden) and a 1-mm straight abutment (Multi-unit Abutment, Nobel Biocare, Goteborg, Sweden). For the posterior implants, two types of implant–abutment connections were selected. In the external hexagon connection (EHC) group, we selected implants with a diameter of 4 mm and a length of 18 mm (NobelSpeed^TM^ Groovy, Nobel Biocare). In the internal hexagon connection (IHC) group, the implants with a diameter of 4.3 mm and a length of 18 mm (NobelParallel^TM^ Conical Connection, Nobel Biocare) were used. In both groups, we selected 30° abutments (30° Multi-unit Abutment, Nobel Biocare). Figure 1 provides a visual representation of all components of the all-on-4 assembly in our study.

**Figure 2:**
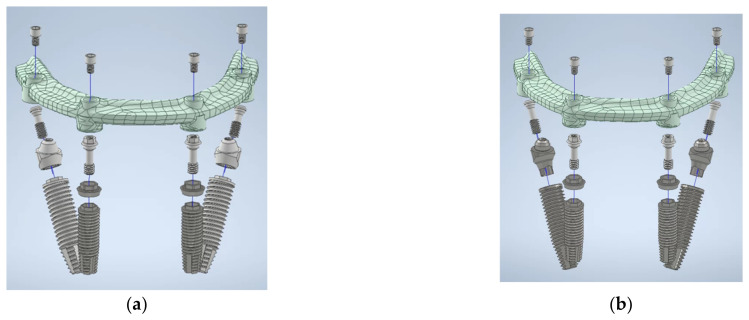
Finite element model in (**a**) the EHC group and (**b**) the IHC group.

All components, including implant bars, MUAs, prosthetic screws, and implant screws, were measured using vernier calipers and a digital microscope to ensure measuring accuracy. To get high-resolution images, the study models were further scanned by a 3D optical scanning system (Aicon SmartScan-HE; Breuckmann, Braunschweig, Germany). Finally, the 3D models were created using computer-aided design (CAD) software (inventor2020; Autodesk, San Rafael, CA, USA) coupled with finite element analysis (FEA) software (ANSYS Workbench 2020 R1; ANSYS, Inc., Canonsburg, PA, USA). The finite element models of the all-on-4 assembly in two groups are shown in Figure 2.

By using finite element analysis (FEA) software (ANSYS Workbench 2020 R1; ANSYS, Inc., Canonsburg, PA, USA), the analyzed model was imported into a bone block model of size 50 mm × 30 mm × 40 mm, which was designed to simulate the structure of human bone. The setting of the bone block model consisted of a 3-mm dense outer layer, which represented the characteristics of cortical bone, and spongy inner content, which replicated cancellous bone.

### 2.2. Finite Element Model Analysis

All components were meshed using tetrahedron elements (SOLID187), specifically, 10-node elements exhibiting quadratic displacement behavior. The SOLID187 is a high-order 3D element that is suitable for modeling irregular meshes. Additionally, to achieve precise results, we used elements of different sizes, ranging from 0.08 to 2.00 mm. The EHC and IHC groups were meshed with a similar number of elements. In the EHC group, approximately 1,885,434 elements and 2,842,741 nodes were used, whereas in the IHC group, 1,948,198 elements and 2,954,778 nodes were used.

The materials used in this study included implant bars, MUAs, prosthetic screws, implant screws, and implant fixtures were verified through energy-dispersive X-ray spectroscopy (JSM-6360; JEOL, Tokyo, Japan). Additionally, for the purposes of our analysis, we assumed that all materials, including bone, exhibited isotropic, homogenous, and linearly elastic characteristics. The mechanical properties of the materials used for the all-on-4 components and bone were identified in previous studies and are listed in Table 1 [27,28,29].

The interface between the cortical bone and cancellous bone was considered to be bonded to enable the primary focus to be on the loading effects on the components of the all-on-4 assembly. The contact area between the implants and bone was assumed to be 100% osseointegrated. Therefore, the interface between the implants and bone was set as bonded. In consideration of frictional effects within each component of the all-on-4 model, we employed a coefficient of friction of 0.3 [30].

**Figure 3:**
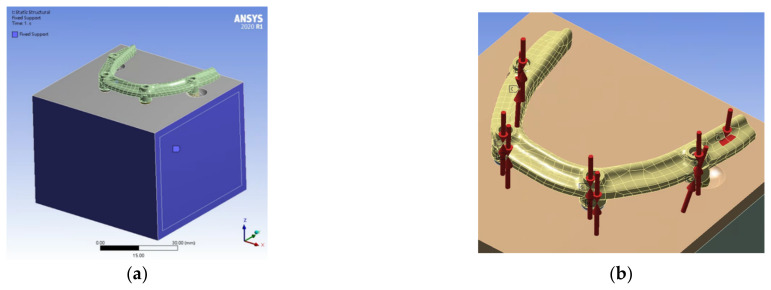
Boundary and loading conditions of the 3D finite element models in our study. (**a**) Boundary condition of a bone block model fixedly supported on all surfaces except the occlusal surface. (**b**) Vertical force of 190 N was applied (arrow A) to the implant bar. Bolt pretension with axial force was noted on the prosthetic screw and implant screws (arrow B–I).

In the boundary conditions, we applied fixed support to all surfaces of the bone block model, restricting displacement in three directions, with the exception of the occlusal surface, to zero (Figure 3a). When applying a force, we converted the tightening torque of the screws into axial force by using the formula [31] T = KDF, where T, K, D, and F represent the tightening torque (N·m), torque coefficient, screw diameter (m), and axial force (N), respectively. In accordance with the manufacturer’s recommendations, we applied the following tightening torques: prosthetic screw, 0.1 N·m; mesial surface of the implant screw, 0.35 N·m; and distal surface of the implant screw, 0.15 N·m. By using these torques, we calculated the corresponding axial force as 192.01, 457.56, and 215.51 N, respectively.

In the loading condition, we applied a vertical force of 190 N on the implant bar, which was positioned approximately 10 mm distal to the prosthetic screw of the posterior implant (Figure 3b).

## 3. Results

### 3.1. Von Mises Stress Values in the EHC and IHC Groups

The groups exhibited similar findings related to stress values and their distributions in the posterior implants of the all-on-4 assembly under identical loading conditions.

First, the maximum von Mises stress values in both groups were primarily those for the prosthetic screws, followed by those for the MUAs. This finding indicates that prosthetic screws and MUAs may represent vulnerable points on the posterior implant within the all-on-4 assembly. Notably, these stress values were close to but did not exceed the yield strength of Ti-6Al-4V alloy, which is approximately 795 MPa. The peak von Mises stress values on the prosthetic screws were 698.00 MPa in the EHC group and 680.52 MPa in the IHC group. Second, the lowest von Mises stress values were consistently observed for bone, with a value of 98.91 MPa being recorded for the EHC group and a value of 93.17 being recorded for the IHC group. This finding indicates that the metallic components within the all-on-4 assembly absorbed a major proportion of the stress, resulting in less stress transfer to the surrounding bone. Additionally, the two groups demonstrated similar magnitudes of von Mises stress on the implant bar, prosthetic screw, MUAs, and bone, with the value of the EHC group being slightly higher than that of the IHC group. Specifically, the peak values of von Mises stress for the implant bar, prosthetic screw, MUAs, and bone in the EHC group were 8.8%, 1.2%, 5.3%, and 6.2% higher than those in the IHC group, respectively. Finally, both groups exhibited a consistent trend of decreasing maximum von Mises stress values from the MUAs to the implant screws.

For the implant screws and implant fixtures, the maximum von Mises stress values were significantly different between the two groups. Notably, the peak stress values for the implant screw and implant fixture in the EHC group were obviously lower than those in the IHC group. The von Mises stress values of the implant screw in the EHC group were 37.75% lower than those in the IHC group. Moreover, the von Mises stress values of the implant fixtures in the EHC group were 33.03% lower than those in the IHC group. These findings indicate that the stress distribution differed in the two groups. In the EHC group, the load transmission was mainly concentrated on prosthetic screws and MUAs, whereas on the implant bar, implant screw, and implant fixture, the stress was evenly distributed with lower von Mises stress values. Conversely, in the IHC group, stress was more evenly distributed in the prosthetic screw, MUAs, implant screw, and implant fixture.

**Figure 4:**
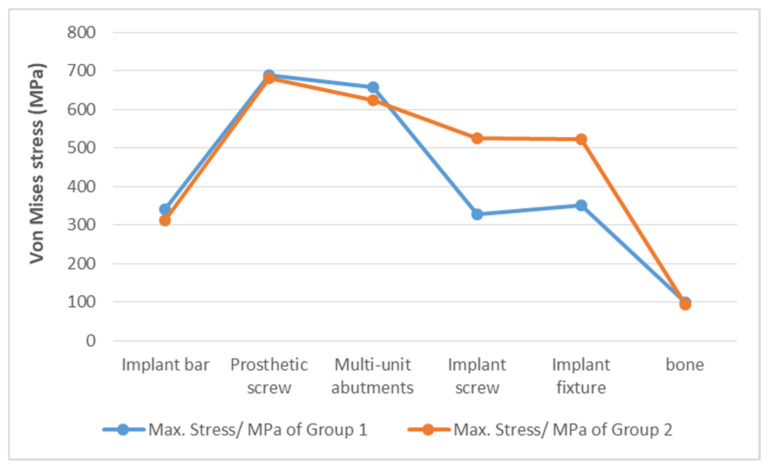
Maximum von Mises stress on each component of the EHC and IHC groups.

Table 2 and Figure 4 present the peak values of the von Mises stress in the two groups.

### 3.2. Stress Distribution Pattern in Each Component of the EHC and IHC Groups

**Figure 5:**
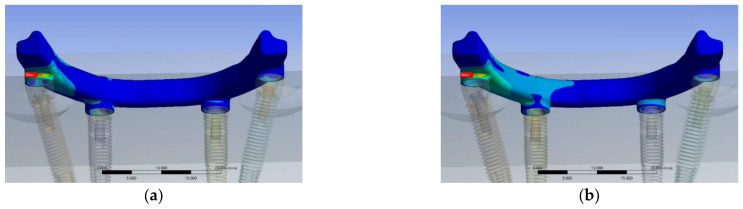

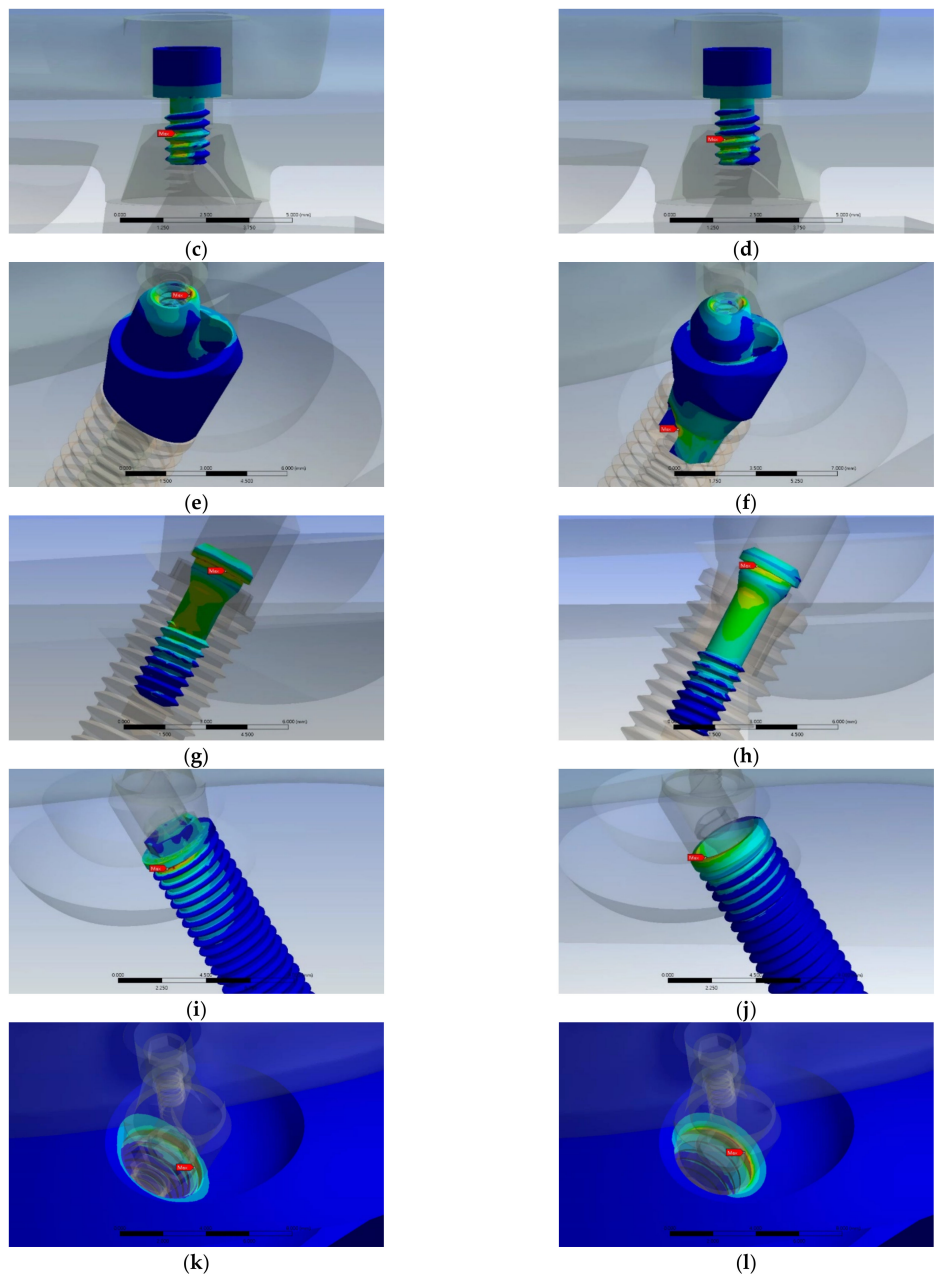
Stress distribution patterns and locations of the maximum von Mises stress for each component of the two groups: Implants bar of the EHC group (**a**) and the IHC group (**b**); prosthetic screws of the EHC group (**c**) and the IHC group (**d**); MUAs of the EHC group (**e**) and the IHC group (**f**); implant screws of the EHC group (**g**) and the IHC group (**h**); implant fixtures of the EHC group (**i**) and the IHC group (**j**); and bones of the EHC group (**k**) and the IHC group (**l**).

The load transmission through each component of the 3D finite element model resulted in different stress distribution patterns. Figure 5 illustrates the stress distribution patterns for the implant bar, prosthetic screw, MUA, implant screw, implant fixture, and bone in both the EHC and IHC groups. Overall, the load force tended to be concentrated at locations where components were interconnected. As mentioned previously, the groups exhibited comparable magnitudes of von Mises stress on the implant bar, prosthetic screw, MUAs, and bone. However, when taking a look at the location where the maximum stress is concentrated within each component of the groups, we found that the maximum stresses for the implant bar (Figure 5a,b) occurred on the distal-lingual surface of the ring junction of the bar. On the prosthetic screw (Figure 5c,d), the highest stresses were concentrated on the third thread of the screw, which corresponds to the middle portion of the screw. Similarly, on the surrounding bone (Figure 5k,l), the positions of maximum stresses for the two groups were on the cervical third of the implant fixture, close to the cantilever region. However, the positions of the peak stress values for the MUAs (Figure 5e,f) were not identical between the EHC and IHC groups. In the EHC group, the peak stress occurred on the inner surface of the distal thread region, which connects with the prosthetic screw. Conversely, in the IHC group, the position of maximum stress was at the junction with the implant fixture.

For the implant screws and implant fixtures in both groups, although the magnitudes of von Mises stress significantly differed, some similarities were noted in the most stressed area. On the implant screws (Figure 5g,h), the maximum stress was observed on the first thread, which connects to the MUAs. Regarding implant fixtures (Figure 5i,j), in both groups, the location of maximum stress was observed on the cervical portion where they connect to the MUAs. In the EHC group, this location was on the external surface of the first thread of the implant fixture. By contrast, in the IHC group, the maximum stress was positioned on the internal contact surface between the MUAs and the implant fixture.

## 4. Discussion

The type of implant–abutment connection plays a crucial role in load transmission, which in turn affects bone remodeling [32]. Implant failure can occur due to either the primary factor, which results in failed osseointegration, or a secondary factor attributable to marginal bone loss. These factors encompass various dimensions, including local, systemic, surgical, and prosthetic considerations [33]. Although implant–abutment connection systems may not be risk factors directly leading to implant failure, they exert a significant influence on the incidence of mechanical and biological complications [34]. In the case of edentulous patients who have undergone reconstruction with an implant-supported prosthesis, the forces generated during mastication often result in excessive stress on the distal implants and their surrounding bone [35].

The manner in which load is transferred and its impact on the stress distribution between dental implants and the surrounding bone have been the focal points of studies involving FEA. Additionally, FEA has proven valuable for assessing and enhancing the biomechanical performance of multi-implant prosthetic treatments, including all-on-4 treatment [11]. FEA is a robust approach to simulating several parameters within a design model, enabling investigation of their potential relevance in real-world clinical scenarios.

In the present study, we applied FEA to investigate the load transmission in the region of the posterior implants within the all-on-4 assembly. Our investigation revealed that the stress distribution patterns differed between the EHCs and IHCs. In the EHC group, the loading force was mainly focused on the prosthetic screws and MUAs. By contrast, in the IHC group, stress was not only distributed more evenly but also slightly lower on the implant bar, prosthetic screw, MUAs, and bone. These findings are consistent with those of prior research that has highlighted the influence on stress distribution with different implant–abutment connection systems [13] and the superior performance of internal connections with respect to stress distribution [16,36].

According to a systemic review and meta-analysis [37], internal connections are favored by the mismatched implant-abutment platform because they contribute to reduced marginal bone loss. In addition, another study [38] explains that the reason why the internal hexagon connections could generate lower stress in bone may be associated with sliding in the tapered joint between the implant and abutment, which can minimize the bending effect.

However, in the present study, the differences in stress distribution values between the implant bars, prosthetic screws, MUAs, and bone in the two groups did not appear to be significant. In the literature, which implant–abutment connection exhibits superior stress distribution remains a topic of debate. Both external and internal connections were demonstrated to exhibit comparable stress distribution in Astrand’s study [39]. Another in vitro study indicated that both systems yielded satisfactory results [15].

Although the IHC group exhibited a more even distribution of stress compared with the EHC group, the values of the maximum von Mises stress on the implant screw and implant fixture in the IHC group were higher than those in the EHC group. Our results indicate that compared with those of the IHC group, the peal values of von Mises stress on the implant screw and the implant fixture in the EHC group were 37.75% and 33.03% lower, respectively. These findings may not be consistent with those of other studies that have focused on stress distribution with different implant–abutment connections in the context of single implants, in which IHCs often exhibit lower stress in the implant neck area than EHCs do [40,41]. Nevertheless, a biomechanical study revealed greater stress concentrations along the implant when it is internally connected [36]. Similar results were reported in a study by Goiato et al. [10], where the EHC in a 3-unit implant-supported prosthesis generated lower stress than the internal connection. These findings may be attributable to factors such as the presence of parallelism among the implants, which can be challenging to achieve when multiple implants are splinted together. This lack of parallelism can influence stress distribution in the case of internal connections. Consequently, the author has recommended opting for EHCs when multi-unit implants are applied.

In the present study, the notable difference in maximum stress values between the MUAs and implant screws in the EHC group, where the implant screw exhibited a 50% lower value compared with that of the MUAs, can be attributed to the distinct stress distribution pattern observed in the MUAs. In the EHC group, the stress was primarily concentrated on the contact area between the MUAs and the prosthetic screw. In the IHC group, although the maximum stress value was slightly lower than that in the EHC group, it occurred at the junction with the implant fixture. The geometrical discontinuity observed in the upper part of the MUAs in the EHC group may have contributed to the stress concentration in this region, leading to a load distribution that affected the load transmission and generated lower stress on the implant screw and implant fixture. This may have prevented the implant screw and implant fixture from loosening and fracturing to some extent. However, this placement in the IHC group may also have increased the risk of mechanical complications occurring in the lower part of the MUAs, where they connect with the implant screws, rather than in the upper portion of the MUAs, where they interact with the prosthetic screws.

Regardless of the implant–abutment system used, the highest maximum von Mises stress values in the two groups were consistently observed for the prosthetic screw, followed by the MUAs. This finding underscores the fact that when an axial force is applied to the distal cantilever region of the mandibular all-on-4 assembly, prosthetic screws and MUAs bear the greatest loading on the posterior implant within the all-on-4 assembly and are, therefore, the components most susceptible to stress. These components are also the ones closest to the yield strength of Ti-6Al-4V alloy, raising concerns regarding potential mechanical complications, such as prosthetic screw fractures and abutment fractures. However, this situation may mitigate more severe mechanical complications that could affect the implant screws or implant fixtures. Our findings align with those of a previous study conducted by Chang et al., which revealed that the primary stresses were concentrated in the abutments, irrespective of whether they were external or internal connections [42]. Similar findings were reported in another FEA study, where the maximum von Mises stress in both internal hexagonal and conical connections occurred in the neck portion of the abutment–prosthesis complex [43].

Our results indicate that the most stressed areas of each component were typically located at the joining parts. However, the positions of peak stress on the MUAs and implant fixtures were not consistent between the two groups. A related study [44] investigated the stress distribution patterns in various implant–abutment systems for single implants and noted differences in the positions of the most stressed areas within the abutment and implant fixture. However, for abutment, they found that the most stressed area in the external connection model was the contact surface with the implant screw, whereas, in the internal connection model, the highest stress was observed in the upper part of the abutment, where a screw seat was designed close to the loaded region. These variations in stress distribution patterns in each component of the implant assembly in our study and the aforementioned studies may be attributable to differences in several factors, including loading conditions, model geometry, and material properties.

A literature review indicated that numerous factors could influence load transfer [11]. To ensure that the influence of connection types would be the primary focus of our analysis, we used implants with the same diameter and length for the anterior implants and implants with the same length and tilting angulation and similar diameters for the posterior implants. Furthermore, to ensure the reliability of the present FEA study and accurate modeling of the stress distribution patterns within each component of the all-on-4 assembly, several fundamental parameters were considered. First, because the primary focus of our study was the components of the all-on-4 assembly, although bone tissue is a heterogeneous structure with marked anisotropy, we applied the simplifying assumption that both cortical and cancellous bone were isotropic, homogenous, and linearly elastic. This simplification enabled us to ensure that the mechanical performance of the bone would be consistent regardless of the direction of the force applied. Another key aspect of our modeling approach was the assumption of perfect osseointegration, where the contact surface between the bone and the implant was set as bonded. Moreover, to prevent any inaccuracies or loosening between the implant fixture and MUAs, we applied tightening torques to the implant screw and prosthetic screw in accordance with the manufacturer’s recommendations. For the loading condition, we simulated masticatory forces on the molar region of the dentition to investigate stress distribution patterns in the two types of implant–abutment connections. A study by Gibbs et al. [45] indicated that in dentate patients, the mean masticatory force is approximately 40% of the maximum biting force during chewing and swallowing. Additionally, a study [46] revealed that the maximum biting force in the right molar region was 513 N among men and 455 N among women. In the left molar region, it was 554 N among men and 443 N among women. Moreover, the study reported that penetration force varies with the type of food being cut; the forces required for rye bread, raw carrot, and boiled meat were reported to be 167, 118, and 80 N, respectively [47]. Considering these factors, we applied a vertical force of 190 N in our study, which is within the range of 40% of the mean maximum biting force and is capable of simulating food penetration during chewing.

This study has several limitations that must be acknowledged. First, although FEA has been demonstrated to be a valuable tool for evaluating the biomechanical performance of dental implant systems, several inherent limitations of this method, including some assumptions and simplifications, may lead to such analysis not accurately reflecting the clinical situation. For example, in our study, we assumed that the bone tissue was isotropic, homogenous, and linearly elastic, which simplified the modeling process but may not have fully captured the complexity of real bone tissue. Some studies [48,49] have employed linear elastic orthotropic models to better approximate the mechanical behavior of bone tissue. Additionally, Pietroń et al. [50] reported that future advancements in bone modeling using tomography and optimization techniques may allow for more realistic representations of bone properties. Several other assumptions and simplifications in this study additionally may not align with real-world conditions. This study did not model a crown prosthesis with specific surfaces set as fixed support in the boundary conditions. Moreover, the loading condition applied in this study included only a single static vertical force, which may not fully simulate the complex biomechanics of mastication. Mastication is a biomechanical process influenced by factors such as dentition, bite force, salivary flow, and jaw muscle activity [51]. Notably, the distribution of strains under vertical or lateral loading was reported to behave differently in EHC and IHC groups [40]. Additionally, under lateral loads, external hexagonal connections may exhibit micro-gaps at the abutment–implant interface [9]. Future studies should consider applying different loading conditions, including horizontal and inclined forces, to provide a more realistic representation of the biomechanical challenges related to dental implant systems during mastication.

Although the solution of FEA can be affected by assumptions and simplifications, which can lead to results not accurately reflecting actual clinical conditions, the parameters and settings used in the current study are computationally acceptable. The results obtained from the FEA can provide valuable information for clinical all-on-4 treatment. The present FEA study revealed that the implant–abutment connection system influenced load transmission and the resulting stress concentration in the posterior implants of the mandibular all-on-4 assembly. Because overloading areas are associated with mechanical complications, such as screw loosening, screw fracture, and abutment fracture [9,10], implant–abutment connections that exhibit significantly lower stress levels should be selected to mitigate the risk of overloading. Additionally, selecting connections where the maximum stress occurs in the safe zone is crucial for avoiding complex mechanical complications. However, for both types of implant–abutment connections, an optimized implant design should be employed to prevent overloading and its associated clinical complications. Further investigations with improved simulation techniques are warranted to better understand the biomechanical performance of these systems. Moreover, clinical studies are needed to prove the findings of the current FEA.

## 5. Conclusions

Based on the findings of this FEA study, the following conclusions can be drawn within the limitations of the research. When an axial force is applied to the distal cantilever region of the mandibular all-on-4 assembly:

1. Both EHCs and IHCs are clinically durable under the tested loading conditions.
2. The most stressed region in the EHC and IHC groups was the prosthetic screw, followed by the MUAs, which indicates these components can be the weakest points on the posterior implant within the all-on-4 assembly.
3. The peak stress value of the implant screw and implant fixture in the EHC group were 37.75% and 33.03% lower than the IHC group, respectively.

Optimizing implant design in conjunction with specific implant–abutment connections is crucial to prevent overload situations and the resulting clinical complications. Additional studies are warranted to validate the findings obtained from the current FEA.

## Figures and Tables

**Table 1:** Mechanical properties of the materials used in the 3D finite element model.

Material	Young’s Modulus (GPa)	Poisson’s Ratio	Yield Strength/MPa
Cortical bone	13.4 [27]	0.30	-
Cancellous bone	1.37 [27]	0.30	-
Pure Titanium (Implant fixture)	115 [28]	0.35	680
Ti-6Al-4V alloy (Implant bar, Implant screw, Prosthetic screw, Screws, Abutments)	110 [29]	0.33	795

**Table 2:** Peak values of von Mises stress on each component in the EHC and IHC groups.

Component	Max. Stress/MPa of the EHC Group	Max. Stress/MPa of the IHC Group
Implant bar	340.29	312.81
Prosthetic screw	689.00	680.52
MUA	657.39	624.37
Implant screw	326.89	525.13
Implant fixture	350.66	523.63
Bone	98.91	93.17

## Data Availability

Correspondence and requests for materials should be addressed to A.Y.-J.W.
